# Additional cytogenetic features determine outcome in patients allografted for *TP53* mutant acute myeloid leukemia

**DOI:** 10.1002/cncr.34268

**Published:** 2022-05-25

**Authors:** Justin Loke, Myriam Labopin, Charles Craddock, Jan J. Cornelissen, Hélène Labussière‐Wallet, Eva Maria Wagner‐Drouet, Gwendolyn Van Gorkom, Nicolaas P.M. Schaap, Nicolaus M. Kröger, Joan Hendrik Veelken, Montserrat Rovira, Anne Lise Menard, Gesine Bug, Ali Bazarbachi, Sebastian Giebel, Eolia Brissot, Arnon Nagler, Jordi Esteve, Mohamad Mohty

**Affiliations:** ^1^ Cancer Research UK, Clinical Trials Unit University of Birmingham Birmingham UK; ^2^ Birmingham Center for Cellular Therapy and Transplantation Centre for Clinical Haematology, Queen Elizabeth Hospital Birmingham UK; ^3^ Acute Leukemia Working Party, Paris Study Office European Society for Blood and Marrow Transplantation Paris France; ^4^ Haematology Department AP‐HP, Saint Antoine Hospital Paris France; ^5^ INSERM, Centre de Recherche Saint‐Antoine (CRSA) Sorbonne Universités, UPMC Univ Paris 06 Paris France; ^6^ Department of Hematology, Erasmus MC Cancer Institute University Medical Center Rotterdam Rotterdam The Netherlands; ^7^ Hopital‐Lyon Sud, Hospices Civils de Lyon, Pierre Bénite France; ^8^ Department of Hematology, Oncology, and Pneumology University Medical Center Mainz Mainz Germany; ^9^ Department Internal Medicine Hematology/Oncology University Hospital Maastricht Maastricht The Netherlands; ^10^ Radboud University Medical Centre Nijmegen The Netherlands; ^11^ University Hospital Eppendorf Bone Marrow Transplantation Centre Hamburg Germany; ^12^ Leiden University Hospital BMT Centre Leiden Leiden The Netherlands; ^13^ Hospital Clinic, Department of Hematology IDIBAPS Barcelona Spain; ^14^ Centre Henri Becquerel, Hematology Rouen France; ^15^ Department of Medicine Goethe University Frankfurt Frankfurt Main Germany; ^16^ Bone Marrow Transplantation Program, Department of Internal Medicine American University of Beirut Medical Center Beirut Lebanon; ^17^ Department of Anatomy, Cell Biology and Physiological Sciences American University of Beirut Beirut Lebanon; ^18^ Maria Sklodowsk‐Curie Memorial Cancer Centre and Institute of Oncology Gliwice Poland; ^19^ Hematology Division, Chaim Sheba Medical Center, Tel Hashomer Ramat Gan Israel

**Keywords:** acute myeloid, leukemia, allogeneic stem cell transplant, cytogenetics, leukemia, *TP53*

## Abstract

**BACKGROUND:**

The presence of *TP53* mutations is associated with an unfavorable outcome in patients allografted for acute myeloid leukemia (AML), leading some to question the benefit of an allogeneic stem cell transplantation (allo‐SCT) for this patient group, although this has not been studied in a large cohort.

**METHODS:**

A total of 780 patients with AML in first complete remission, with either intermediate‐ or adverse‐risk cytogenetics, whose *TP53* mutation status was reported, were included in this study from the European Society for Blood and Marrow Transplantation.

**RESULTS:**

Two‐year overall survival (OS) was impaired in patients (n = 179) with evidence of a *TP53* mutation at diagnosis (35.1%; 95% confidence interval [CI], 26.7–43.7) as compared to the cohort without (n = 601) (64%; 95% CI, 59.1–68.4; *P* = .001). In patients with mutant *TP53* AML with no evidence of either chromosome 17p loss (17p–) and/or complex karyotype (CK) (n = 53, 29.6%), 2‐year OS was 65.2% (95% CI, 48.4–77.6). This was not significantly different to patients without *TP53* mutations. In patients with mutant *TP53* AML with either 17p– and/or CK (n = 126, 70.4%), the OS was lower (24.6%; 95% CI, 16.2–34; *P* = .001).

**CONCLUSIONS:**

In summary, the adverse prognostic effect of *TP53* mutations in AML following an allo‐SCT is not evident in patients with neither co‐occurring 17p– and/or CK, and these data inform decisions regarding allo‐SCT in patients with *TP53* mutant AML.

## INTRODUCTION

Allogeneic stem cell transplantation (allo‐SCT) provides a potentially curative treatment for patients with acute myeloid leukemia (AML) due to an ability to deliver both intensified chemotherapy and a potent graft‐versus‐leukemia effect.^1^ This results in a reduction in risk of disease relapse even in groups of patients with adverse prognostic features.^2^ The decision to proceed to an allograft is predicated on a number of factors including the reduction in risk of relapse following an allo‐SCT.^3^ The incorporation of genetic mutations have provided further powers of refinement to risk of relapse without a transplant,^4^ but evidence for their prognostic value after an allo‐SCT, individually and in association with other cytogenetic features, is currently limited by the relatively small cohorts of patients studied so far.

*TP53* mutations account for approximately 10% of AML cases^4^ and are associated with a dismal prognosis when treated with intensive chemotherapy alone.^5^, ^6^ There is limited data on outcomes for *TP53* mutant AML post allo‐SCT.^7^ Outcomes in *TP53* mutant myelodysplastic syndrome (MDS) after allo‐SCT^7^, ^8^, ^9^ show low survival rates that have led some to question the value of this treatment in patients with *TP53* myeloid malignancies. It is also unknown whether other cytogenetic and genetic features may affect the outcome of patients with *TP53* mutant AML after an allo‐SCT, but is established in the nontransplant setting.^10^, ^11^ Analyses of patients with *TP53* mutant MDS present conflicting results as to the significance of coexisting cytogenetic abnormalities.^12^, ^13^ The presence of *TP53* mutations and coexisting complex karyotype in patients with MDS may lead to reduced overall survival (OS) due to increased relapse rates,^12^ but this has not been replicated in other large studies.^13^ Unravelling the prognostic impact of coexisting cytogenetic abnormalities is of particular importance given the frequent co‐occurrence of *TP53* with these other genetic features associated with increased risk of relapse post allo‐SCT,^6^, ^14^ which include cytogenetic abnormalities leading to loss of 17p region harboring wild‐type *TP53* allele.^15^ It is also unclear whether other variables such as conditioning intensity, graft‐versus‐host disease (GVHD) prophylaxis strategy, or donor selection has any impact on the posttransplant outcomes of patients with *TP53* mutant AML.

The aim of this Acute Leukemia Working Party (ALWP) study from the European Society for Blood and Marrow Transplantation (EBMT), was to elucidate the prognostic value of *TP53* mutations in patients with AML in first complete remission (CR1) that undergo an allo‐SCT and to identify any further determinants of transplant outcomes in this subgroup of patients.

## MATERIALS AND METHODS

### Study Design and Data Collection

We analyzed the outcomes of patients with AML who had intermediate or adverse risk cytogenetic abnormalities, who received an allo‐SCT in CR1 between 2015 and 2019, according to their *TP53* mutation status. Patients were only included in this analysis if their *TP53* mutation status was reported to the EBMT registry. There was no restriction to inclusion with regards to age, conditioning intensity, or donor source. Monosomal karyotype was defined as per previous studies,^16^ and complex karyotype is defined as 3 or more structural cytogenetic abnormalities.^17^ Cytogenetic risk has been defined previously.^18^ Abnormalities of chromosome 17 were defined as any abnormalities, including −17 or del(17p). Cytogenetic and genetic results were from diagnosis. Measurable residual disease (MRD) monitoring were performed by contributing centers.^19^

The review committee of the ALWP of the EBMT registry approved and provided the data for this study. The EBMT is a voluntary group of more than 600 transplant centers that are required to report consecutive stem cell transplantations and follow‐ups once a year (Supporting Table 1). Patients participating in this study provided informed consent for their data to be used in this registry study.

### Statistical Analysis

Patient‐, disease‐, and transplant‐related characteristics for the 2 cohorts (*TP53* mutation, present vs absent) were compared by using χ^2^ statistics for categorical variables and the Mann–Whitney test for continuous variables. Acute and chronic GVHD was graded as per previous definitions.^19^, ^20^ Relapse was defined as an increase of 5% blasts or more. Nonrelapse mortality (NRM) was defined as death without relapse. OS and leukemia‐free survival (LFS) were defined from time of transplant to the event. NRM, cumulative incidence of relapse (CIR), and acute and chronic GVHD were estimated using cumulative incidence to accommodate for competing risks. In the study of acute and chronic GVHD, relapse and death were defined as competing events. The Kaplan–Meier method was used to estimate the probabilities of LFS and OS.^21^, ^22^ Gray's test for cumulative incidence functions and the log‐rank test for OS and LFS were used in univariate analyses. All variables differing significantly between the 2 groups, or factors known to be associated with the outcome after transplantation, were included in a Cox proportional hazards model. We also performed a multivariate analysis of patients with *TP53* mutant AML to identify factors which might influence posttransplant outcomes in this group. In the Cox model, we included all major variables (patient age, de novo vs secondary AML, patient and donor cytomegalovirus seropositivity, and use of posttransplant cyclophosphamide [PTCy]) and performed a stepwise selection of cytogenetics abnormalities (del 5q/−5, del17p, and complex karyotype). Results were expressed as the hazard ratio (HR) with a 95% confidence interval (95% CI). Two‐sided tests were used. To determine factors associated with time‐to‐event outcomes, a type 1 error rate was fixed at 0.05. Analyses were performed using R 4.1.1.^23^

## RESULTS

### Patient and Transplant Characteristics

A total of 601 patients without a *TP53* mutation and 179 patients with a *TP53* mutation were identified. Although this percentage of *TP53* mutations is higher than that found in newly diagnosed patients,^4^ it is consistent with previous studies incorporating genomic profiling in cohorts of patients with MDS or AML undergoing allo‐SCT,^13^, ^24^ The median follow‐up period was 18 months. The median age of the entire cohort was 58 years. Patient, transplant, and disease characteristics according to the presence or absence of *TP53* mutations are described in Table 1. There were no differences between the groups in terms of conditioning intensity, donor source, or T‐cell depletion. There was a slight increase in age and in frequency of secondary AML in patients with a *TP53* mutation as compared to those without this abnormality.

**TABLE 1:** Patient Characteristics According to Presence of *TP53* Mutations.

		*TP53* Mutation		*P*
		Absent (n = 601)	Present (n = 179)	
Patient age (y)	Median (min‐max)	57.6 (18.3–77.4)	60.2 (22.6–75.7)	.043
Type of AML	Primary	524 (87.2%)	145 (81%)	.038
	Secondary	77 (12.8%)	34 (19%)	
Karyotype	Normal	336 (55.9%)	29 (15.7%)	<.0001
	Abnormal	265 (44.1%)	150 (84.3%)	
Abnormal chromosome 17	Absent	578 (96.2%)	111 (62.0%)	<.0001
	Present	23 (3.8%)	68 (38.0%)	
Del(5q)/−5	Absent	573 (95.3%)	92 (51.4%)	<.0001
	Present	28 (4.7%)	87 (48.6%)	
Del(7q)/−7	Absent	554 (92.2%)	119 (66.5%)	<.0001
	Present	47 (7.8%)	60 (33.5%)	
Complex karyotype	Absent	531 (88.4%)	63 (35.2%)	<.0001
	Present	70 (11.6%)	116 (64.8%)	
Monosomal karyotype	Absent	551 (91.7%)	106 (59.2%)	<.0001
	Present	50 (8.3%)	73 (40.8%)	
MRC classification	Intermediate	443 (73.7%)	36 (20.1%)	<.0001
	Poor	158 (26.3%)	143 (79.9%)	
Type of donor	Fully matched sibling	159 (26.5%)	37 (20.7%)	.28
	Unrelated	350 (58.2%)	114 (63.7%)	(MSD vs UD vs other)
	Other relative	69 (11.5%)	24 (13.4%)	
	Cord	23 (3.8%)	4 (2.2%)	
Conditioning intensity	MAC	256 (42.7%)	61 (34.9%)	.064
	RIC	344 (57.3%)	114 (65.1%)	
	Unknown	1	4	
Karnofsky score	<90	136 (23.3%)	48 (28.4%)	.17
	≥90	448 (76.7%)	121 (71.6%)	
	Unknown	17	10	
In vivo T‐cell depletion	Absent	286 (48.1%)	80 (44.9%)	.45
	Present	308 (51.9%)	98 (55.1%)	
	Unknown	7	1	
PTCy	No PTCy	438 (74.6%)	136 (77.3%)	.47
	PTCy	149 (25.4%)	40 (22.7%)	
	Missing	14	3	

Abbreviations: AML, acute myeloid leukemia; complex karyotype, 3 or more abnormalities^17^; cytogenetic risk^18^; MAC, myeloablative conditioning; max, maximum; min, minimum; monosomal karyotype, 2 or more autosomal monosomies or a single autosomal monosomy plus an additional structural characteristic^16^; MRC, Medical Research Council; MSD, matched sibling donor; PTCy, Posttransplant cyclophosphamide; RIC, reduced intensity conditioning; UD, unrelated donor.

In patients with TP53 mutation, only 16% harbored a normal karyotype. In contrast, there was a highly statistically significant enrichment of chromosomal abnormalities in chromosomes 17p (31% vs 3%), 5q (49% vs 5%), and 7q (34% vs 8%) as well as complex (65% vs 12%) and monosomal karyotypes (41% vs 8%) in the cohort of patients with mutated *TP53* in comparison to those without (all comparisons, *P* < .05), which is consistent with previous observations.^4^ The frequency of complex karyotype in the *TP53* mutant subgroup is lower than in previous reports of newly diagnosed patients with *TP53* mutant AML^10^ and may reflect a subgroup of patients who may arrive at an allograft in a CR.

### *TP53* Mutations Result in Increased Risk of Relapse and Reduced OS and LFS in Patients With AML Following an Allo‐SCT

**FIGURE 1:**
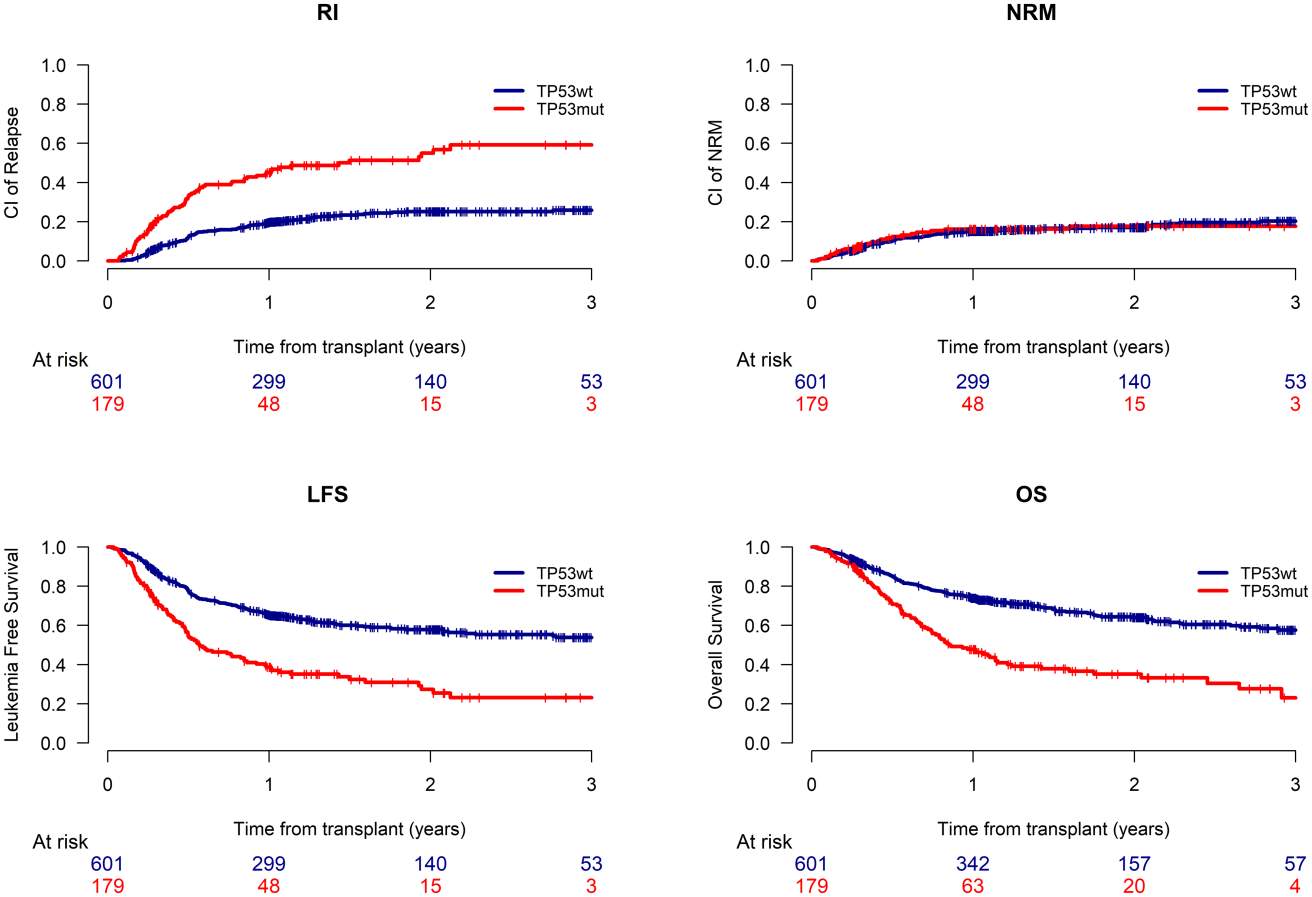
(A) RI, (B) NRM, (C) LFS, and (D) OS from time of transplant for patients with AML with or without mutations at *TP53* post allogeneic stem cell transplantation. AML indicates acute myeloid leukemia; LFS, leukemia‐free survival; NRM, nonrelapse mortality; OS, overall survival; RI, relapse incidence.

The effect of *TP53* mutation on transplant outcome was first examined by performing a univariate analysis, results of which are shown in Supporting Table 2. Patients with a *TP53* mutation had an increased CIR of 55% (95% CI, 45.2–63.8) at 2 years, in comparison to those without, who had a CIR of 25.2% (95% CI, 21.2–29.3; *P* = .001) (Fig. 1; Supporting Table 2). This resulted in an inferior OS at 2 years of 35% (95% CI, 26.7–43.7) for patients with a *TP53* mutation as compared to 64% (95% CI, 59.1–68.4; *P* = .001) in those without a mutation in *TP53*. *TP53* mutations had no impact on rates of NRM but LFS at 2 years was also significantly reduced in the presence of a *TP53* mutation (*TP53* mutation present: 27.3%, 95% CI, 19–36.3; no *TP53* mutation: 57.8%, 95% CI, 53.1–62.3; *P* = .001). Other adverse‐risk cytogenetic subgroups such as complex and monosomal karyotypes, loss of 17p (−17 or del(17p)), del(7q), and del (5q) increased risk of relapse after allo‐SCT in this cohort and similarly resulted in a statistically significantly reduced OS (Supporting Table 2).

**TABLE 2:** Cox Multivariate Analysis of *TP53* Mutations and Other Patient‐ and Transplant‐Related Variables Post Allo‐SCT

	Relapse		NRM		LFS		OS		Acute GVHD II‐IV		Chronic GVHD	
	HR (95% CI)	*P*	HR (95% CI)	*P*	HR (95% CI)	*P*	HR (95% CI)	*P*	HR (95% CI)	*P*	HR (95% CI)	*P*
*TP53* mutation	1.74 (1.19–2.54)	.004	1.07 (0.62–1.87)	.8	1.46 (1.07–1.98)	.018	1.49 (1.07–2.08)	.017	1.03 (0.7–1.52)	.88	1.12 (0.74–1.69)	.59
Abnormal 17p	1.35 (0.87–2.1)	.19	1.36 (0.64–2.9)	.43	1.34 (0.91–1.96)	.13	1.44 (0.96–2.15)	.077	1.15 (0.68–1.95)	.59	0.89 (0.47–1.67)	.71
Del(5q)/−5	1.84 (1.18–2.89)	.008	1.23 (0.61–2.48)	.55	1.64 (1.13–2.39)	.009	1.62 (1.09–2.41)	.017	1.16 (0.7–1.93)	.56	0.26 (0.13–0.52)	.0001
Del(7q)/−7	0.85 (0.55–1.31)	.46	1.12 (0.59–2.12)	.73	0.92 (0.64–1.33)	.67	0.9 (0.62–1.31)	.58	1.16 (0.73–1.84)	.53	1.64 (0.99–2.72)	.056
Complex karyotype	1.73 (1.15–2.6)	.009	1.19 (0.66–2.15)	.56	1.48 (1.06–2.06)	.02	1.55 (1.09–2.21)	.014	1.09 (0.72–1.65)	.68	1.72 (1.16–2.56)	.007
Monosomal karyotype	1.2 (0.78–1.85)	.4	0.59 (0.28–1.22)	.15	1 (0.69–1.43)	.99	0.79 (0.53–1.18)	.25	0.73 (0.45–1.18)	.2	0.82 (0.5–1.36)	.45
Age (per 10 y)	0.98 (0.87–1.11)	.77	1.54 (1.25–1.9)	<.0001	1.12 (1.01–1.25)	.031	1.23 (1.09–1.38)	.0006	1.01 (0.9–1.13)	.91	0.92 (0.82–1.04)	.17
Donor matched sibling donor (reference)	1		1		1		1		1		1	
Unrelated vs matched sibling donor	0.76 (0.54–1.07)	0.12	2.1 (1.19–3.7)	.011	1.09 (0.82–1.45)	.58	1.19 (0.87–1.61)	.27	1.14 (0.82–1.58)	.44	1.02 (0.74–1.42)	.89
Other donor	0.83 (0.51–1.34)	0.45	2.72 (1.4–5.3)	.003	1.24 (0.85–1.81)	.26	1.28 (0.85–1.93)	.23	1.04 (0.67–1.63)	.86	0.88 (0.56–1.39)	.58
secondary AML vs de novo AML	1.28 (0.88–1.87)	0.2	1.35 (0.84–2.16)	.22	1.34 (1–1.8)	.054	1.17 (0.84–1.62)	.36	1.07 (0.72–1.59)	.73	1.19 (0.82–1.73)	.37
Female‐to‐male	0.69 (0.45–1.06)	0.086	1.47 (0.93–2.33)	.096	0.98 (0.72–1.33)	.87	1.04 (0.75–1.44)	.82	1.27 (0.9–1.77)	.17	1.52 (1.08–2.16)	.018
Recipient CMV‐positive	0.8 (0.58–1.1)	0.17	1.81 (1.14–2.87)	.013	1.1 (0.85–1.43)	.45	1.28 (0.97–1.7)	.086	0.96 (0.71–1.29)	.77	1.16 (0.85–1.59)	.35
Donor CMV‐positive	0.91 (0.67–1.25)	0.58	0.77 (0.52–1.13)	.18	0.82 (0.65–1.05)	.12	0.81 (0.62–1.05)	.12	0.84 (0.63–1.11)	.22	1 (0.75–1.33)	.98
RIC vs MAC	0.76 (0.55–1.06)	0.1	0.87 (0.57–1.34)	.53	0.81 (0.63–1.05)	.11	0.81 (0.62–1.07)	.14	0.84 (0.62–1.12)	.23	1.35 (0.99–1.83)	.055

Abbreviations: AML, acute myeloid leukemia; CI, confidence interval; CMV, cytomegalovirus; GVHD, graft‐versus‐host disease; HR, hazard ratio; LFS, leukemia‐free survival; MAC, myeloablative conditioning; NRM, nonrelapse mortality; OS, overall survival; RIC, reduced intensity conditioning; SCT, stem cell transplantation.

Given the increased co‐occurrence of *TP53* mutations with these other cytogenetic abnormalities associated with an increased risk of relapse, we performed a Cox regression analysis to determine the independent risk of *TP53* mutations in patients with AML following an allo‐SCT (Table 2). When adjusting for other variables that significantly affected posttransplant outcomes, mutations in *TP53* remained an important determinant of relapse risk (adjusted hazard ratio [HR], 1.74; 95% CI, 1.19–2.54; *P* = .004) and OS and LFS remained impaired at 2 years (adjusted HR for OS, 1.49; 95% CI, 1.07–2.08; *P* = .017 and adjusted HR for LFS: 1.46; 95% CI, 1.07–1.98; *P* = .018).

In this multivariate analysis, the other factors that increased relapse risk and reduced OS in this cohort of AML patients with intermediate and adverse risk cytogenetics who received an allo‐SCT in CR1 were the presence of either deletion or loss of chromosome 5 (adjusted HR, 1.62; 95% CI, 1.09–2.41; *P* = .017) and the presence of a complex karyotype (adjusted HR, 1.55; 95% CI, 1.09–2.21; *P* = .014). Moreover, increasing age was associated with a higher NRM risk (HR, 1.54 per 10‐year increased; 95% CI, 1.25–1.9; *P* < .0001) and shorter OS (adjusted HR, 1.23; 95% CI, 1.09–1.38; *P* = .0006).

### Chromosome 17p Loss or Complex Karyotype Increase Risk of Relapse in Patients With *TP53* Mutant AML and Reduce Overall Survival

Given the striking increase in disease risk identified for patients with *TP53* mutant AML, we sought to identify factors in this cohort of patients that might influence posttransplant outcomes. We identified a number of factors associated with a statistically significant increased risk of relapse, with a subsequent reduction in OS (Supporting Table 3). For example, patients with *TP53* mutations in combination with losses at chromosome 17p had a 2‐year CIR of 71.14% (95% CI, 52.7–83.8) and OS of 16.3% (95% CI, 6.9–29.2). In contrast, patients without losses at chromosome 17p had a reduced CIR at 2 years of 47.4% (95% CI, 35.6–58.3; *P* = .007) and improved OS of 44.7% (95% CI, 33.9–55; *P* = .001). Similarly, patients with a complex karyotype had a CIR at 2 years of 66.4% (95% CI, 54.6–75.8) and an OS of 23.1% (95% CI, 14.7–32.8). In contrast, patients without a complex karyotype had an improved CIR of 30.8% (95% CI, 17.2–45.5; *P* = .001) and OS of 61.9% (95% CI, 46.8–73.8; *P* = .001). Neither transplant intensity nor use of in vivo T‐cell depletion impacted relapse risk. The presence of pretransplant MRD was associated with an inferior overall survival (Supporting Table 3). This analysis was limited to the patients in whom this data was available.

**TABLE 3:** Outcomes at 2 Years for Patients With Unmutated *TP53* AML and Mutated TP53 AML and Presence of Abnormalities at Chromosome 17p or Complex Karyotype

		Relapse	NRM	LFS	OS	Acute GVHD II‐IV (at 180 d)	Chronic GVHD
TP53 unmutated (n = 601)		25.2% (21.2–29.3)	17% (13.8–20.5)	57.8% (53.1–62.3)	64% (59.1–68.4)	41.4% (36.7–46)	29.7% (26–33.5)
TP53 mutated: abnormal 17p or complex karyotype	Absent (n = 53)	27.5% (13.4–43.7)	12.5% (4.9–23.8)	59.9% (41.7–74.1)	65.2% (48.4–77.6)	33.2% (20.7–46.3)	31.1% (17.7–45.5)
	Present (n = 126)	65.4% (53.9–74.8)	19.3% (12.1–27.8)	15.2% (8–24.6)	24.6% (16.2–34)	31.7% (23.7–40.1)	24.3% (16.1–33.4)
*P*		.001	.48	.001	.001	.88	.38

Abbreviations: AML, acute myeloid leukemia; GVHD, graft‐versus‐host disease; LFS, leukemia‐free survival; NRM, nonrelapse mortality; OS, overall survival.

We used a Cox proportional hazards regression model to account for other prognostic factors related to patient, donor, and transplant characteristics and co‐occurrence of more than 1 poor prognostic variable (Supporting Table 4). In this analysis, only losses at either chromosome 17p or complex karyotype resulted in a reduction in both OS and LFS in patients with *TP53* mutant AML. The presence of complex karyotype resulted in a reduced OS (adjusted HR, 1.99; 95% CI, 1.06–3.75; *P* = .033) and increased the risk of relapse (adjusted HR, 2.55; 95% CI, 1.25–5.18; *P* = .01). Similarly, chromosome 17p losses was associated with a reduced OS (adjusted HR, 1.71; 95% CI, 1.08–2.71; *P* = .023).

**TABLE 4:** Cox Multivariate Comparison of TP53 Mutated AML With and Without Losses at Chromosome 17p or Complex Karyotype With AML Without TP53 mutations

	Relapse		NRM		LFS		OS		Acute GVHD II‐IV		Chronic GVHD	
	HR (95% CI)	*P*	HR (95% CI)	*P*	HR (95% CI)	*P*	HR (95% CI)	*P*	HR (95% CI)	*P*	HR (95% CI)	*P*
TP53‐unmutated (reference)	1		1		1		1		1		1	
TP53 mutated without abnormal 17p or complex karyotype	1.19 (0.64–2.22)	.57	0.94 (0.41–2.16)	.89	1.08 (0.66–1.77)	.77	1.16 (0.69–1.98)	.57	1.12 (0.68–1.86)	.65	0.93 (0.52–1.63)	.79
TP53 mutated with abnormal 17p or complex karyotype	4.68 (3.41–6.42)	<.0001	1.36 (0.81–2.28)	.24	3.06 (2.36–3.98)	<.0001	2.9 (2.2–3.82)	<.0001	1.13 (0.79–1.62)	.51	0.98 (0.62–1.53)	.91
Age (per 10 y)	0.98 (0.87–1.11)	.79	1.53 (1.24–1.89)	<.0001	1.12 (1.01–1.25)	.031	1.23 (1.09–1.38)	7.00e−04	1.01 (0.9–1.13)	.9	0.91 (0.81–1.03)	.13
Donor MSD (reference)	1		1		1		1		1		1	
UD vs MSD	0.72 (0.51–1.01)	.054	2.1 (1.19–3.69)	.01	1.04 (0.79–1.38)	.77	1.15 (0.85–1.56)	.37	1.13 (0.81–1.56)	.47	1.03 (0.75–1.44)	.84
Other donor	0.71 (0.44–1.14)	.15	2.75 (1.43–5.29)	.003	1.14 (0.79–1.65)	.47	1.17 (0.79–1.75)	.44	1.04 (0.67–1.62)	.85	0.94 (0.6–1.46)	.77
Secondary AML vs de novo AML	1.29 (0.88–1.88)	.19	1.32 (0.82–2.11)	.25	1.31 (0.98–1.77)	.07	1.12 (0.81–1.55)	.5	1.07 (0.72–1.58)	.74	1.19 (0.82–1.72)	.36
Female‐to‐male	0.7 (0.46–1.08)	.11	1.51 (0.96–2.38)	.072	1 (0.73–1.36)	1	1.06 (0.76–1.47)	.74	1.27 (0.91–1.78)	.16	1.51 (1.07–2.13)	.019
Patient CMV pos	0.8 (0.58–1.1)	.17	1.85 (1.16–2.93)	.009	1.09 (0.84–1.41)	.5	1.27 (0.96–1.67)	.099	0.97 (0.72–1.3)	.82	1.15 (0.84–1.57)	.37
Donor CMV pos	0.89 (0.65–1.21)	.45	0.76 (0.52–1.12)	.17	0.82 (0.65–1.05)	.12	0.81 (0.62–1.05)	.11	0.84 (0.63–1.11)	.22	1.04 (0.78–1.38)	.8

Abbreviations: AML, acute myeloid leukemia; CI, confidence interval; CMV, cytomegalovirus; GVHD, graft‐versus‐host disease; HR, hazard ratio; LFS, leukemia‐free survival; MSD, matched sibling donor; NRM, nonrelapse mortality; OS, overall survival; pos, seropositive; PTCy, posttransplant cyclophosphamide; RIC, reduced intensity conditioning; UD, unrelated donor.

**FIGURE 2:**
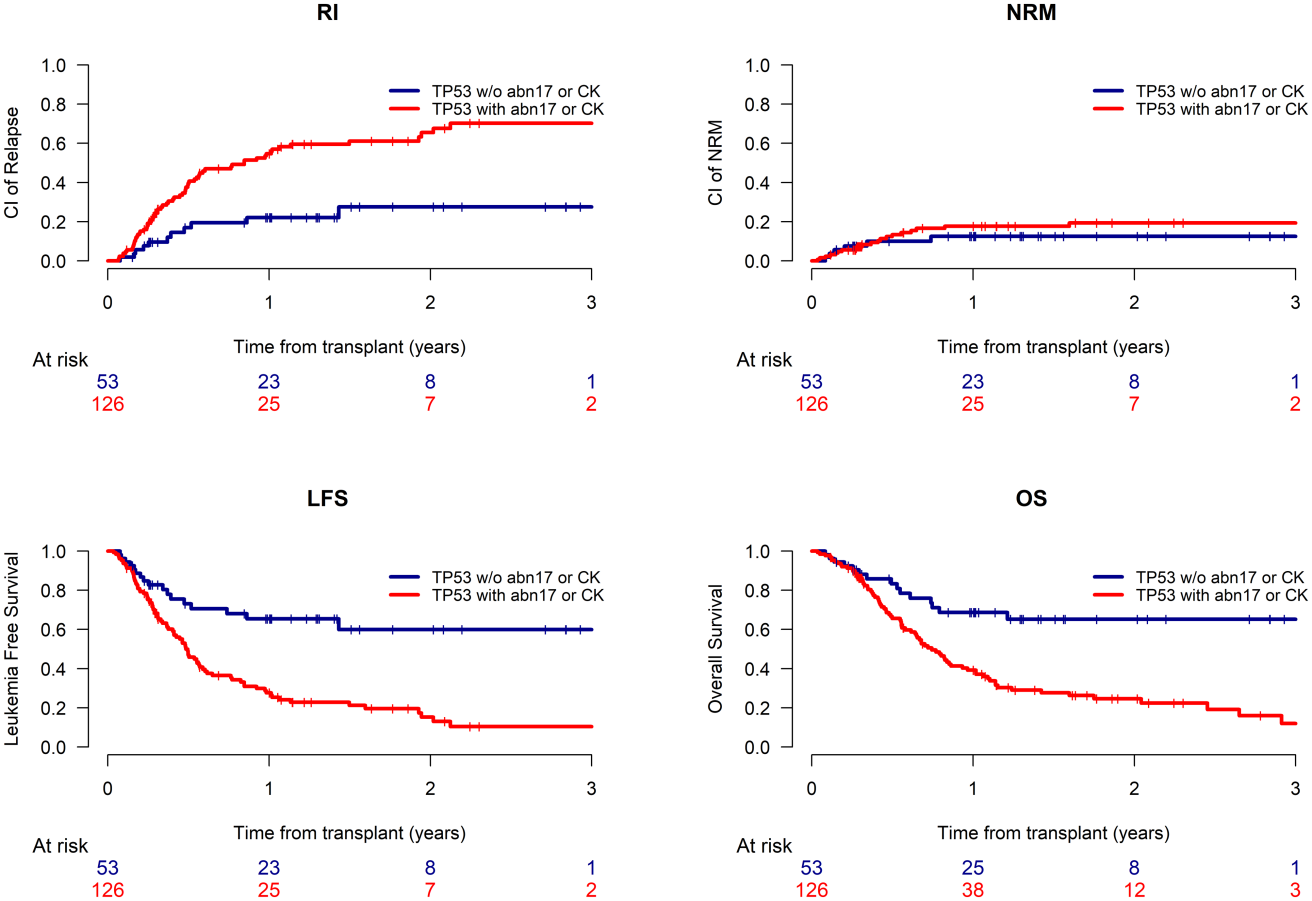
(A) RI, (B) NRM, (C) LFS, and (D) OS for patients with TP53 mutant AML with or without abnormalities at chromosome 17p (−17 or del(17p)) or complex karyotype. AML indicates acute myeloid leukemia; LFS, leukemia‐free survival; NRM, nonrelapse mortality; OS, overall survival; RI, relapse incidence.

As such, we were able to identify a cohort of patients with *TP53* mutations who had either a loss of 17p and/or complex karyotype (n = 126) or neither of these karyotypic abnormalities (n = 53). Patients with *TP53* mutant AML with neither 17p losses and/or complex karyotype had a favorable OS at 2 years of 65.2% (95% CI, 48.4–77.6), with a CIR at 2 years of 27.5% (95% CI, 13.4–43.7). In contrast, in the presence of either or both loss of 17p and/or complex karyotype, the OS at 2 years was only 24.6% (95% CI, 16.2–34; *P* = .001) with a higher CIR of 65.4% (95% CI, 53.9–74.8; *P* = .001) (Fig. 2; Table 3,).

Strikingly, there was no significant difference in risk of relapse or survival between patients with *TP53* mutations, without either loss of 17p and/or complex karyotype, as compared to patients without a *TP53* mutation, even when this was adjusted for other risk factors. Treating patients with AML with unmutated *TP53* as a reference, patients with mutated *TP53* alone had an adjusted HR of 1.16 (95% CI, 0.69–19.98; *P* = .57) for OS. In contrast, patients with mutated *TP53* and either 17p loss and/or complex karyotype had an inferior OS with an adjusted HR of 2.9 (95% CI, 2.2–3.82; *P* < .0001) (Table 4).

## DISCUSSION

In this study from the ALWP of the EBMT, we demonstrate that patients with *TP53*‐mutated AML who have undergone an allo‐SCT in CR1 had an inferior OS to those without a mutation in *TP53*, regardless of the presence of concomitant high risk cytogenetic features, such as loss of chromosome 5 or 7. However, the poor prognosis of patients with mutations at *TP53* seems to be restricted to those patients with concomitant loss of 17p and/or complex karyotypes. Even in this latter subgroup with additional high‐risk features, 2‐year OS was possible in a sizeable cohort of patients, suggesting that allo‐SCT remains the only curative strategy for this group. This is of notable clinical significance due to the ongoing debate surrounding the issue of whether allo‐SCT can provide long‐term survival in patients with *TP53* mutant AML.^25^ Thus, further investigation should revolve around the optimal route to transplant and potential posttransplant maneuvers to prevent relapse, given the high rates of primary refractory disease and high relapse following allo‐SCT in this cohort.^5^

Our data clearly show that a complex karyotype significantly increases the risk of relapse in patients with *TP53* mutant AML posttransplant. Previous large studies have shown varying results for whether a complex karyotype may additionally provide prognostic information on *TP53* mutant MDS following an allograft,^12^, ^13^ in part dependent on the study‐specific technologies used to detect chromosomal abnormalities. The high frequency of complex karyotype among TP53‐mutant AML patients may reflect a higher cytogenetic instability and higher tolerance to gross karyotypic aberrancies in leukemic cells lacking functional wild‐type TP53 protein. The combined poor prognostic impact of CK and TP53 mutations have been observed in AML by a recent study from the Dutch–Belgian Hemato‐Oncology Cooperative group (HOVON).^11^

This study supports the importance of biallelic loss of *TP53* activity in AML following an allograft, as seen in the additional poor prognostic implications of concomitant 17p abnormalities in patients with *TP53* mutant AML. Recently, in a largely nontransplant setting, biallelic loss of *TP53* activity has been shown to be important in MDS,^26^ where biallelic loss of *TP53* activity was inferred through several routes, including variant allelic frequency (VAF). In 2 recent studies, VAF was an independent prognostic factor in predicting response to hypomethylating agents in patients with *TP53* mutant MDS,^27^ and in the context of AML, the impact of *TP53* VAF may be dependent on treatment intensity.^10^ In contrast, 2 further recent studies provide conflicting data: focusing on large newly diagnosed AML cohorts, 2 independent study groups did not find a conclusive link between VAF size,^11^, ^28^ nor presence of biallelic mutations,^11^ with patient survival. None of these studies were sufficiently powered to investigate the interaction between VAF and outcomes following allo‐SCT, and this will be of interest in future studies.

In patients with a *TP53* mutation, lower intensity regimens such as venetoclax‐based regimens may have at least comparable responses to patients treated with intensive treatment, and they may proceed to an allograft in a fitter state.^29^ Further developments in immunotherapy with anti‐CD47 antibody, Magrolimab, may also provide an alternative remission induction or salvage strategy for some patients.^30^ Whether these novel pretransplant therapies affect posttransplant outcomes should be of future investigation. Posttransplant relapse remains the major cause of failure of transplant in this setting, and, as such, strategies to reduce this warrant further investment of effort. Maintenance strategies such as the ongoing trial of oral Azacitidine (CC‐486, clinical trial NCT04173533) may provide options for this group of patients and should be a question for further work.

In this study, it was notable that we did not identify a significant transplant variable, including conditioning intensity, GVHD prophylaxis, or use of T‐cell depletion, which altered the outcomes of patients with *TP53* mutant AML. Recent findings suggest that *TP53*‐mutated AML is associated with an immunosuppressive microenvironment.^31^ This suggests  maneuvers aimed at re‐modelling the immune‐privileged environment, such as PD‐1 blockade,^32^ may restore a graft‐versus‐leukemia effect. A further avenue of future research will be to define the role of pretransplant MRD in predicting outcomes posttransplant in *TP53* mutant AML. Pretransplant MRD is of clear significance in predicting posttransplant outcomes through a range of different methodologies.^23^, ^33^ It is currently unknown whether these technologies may help inform our prediction of posttransplant outcomes in the setting of *TP53* mutant AML.^34^ In our current data, with the caveat that pretransplant MRD results were available for analysis in only a limited number of patients, pretransplant MRD appears to result in inferior overall survival of patients with *TP53* mutant AML. Future studies should address this issue comprehensively. Given the recent data on the impact of conditioning intensity on outcomes of patients with pretransplant MRD,^24^ and the likely high prevalence of MRD positivity pretransplant in this subgroup,^34^ it is of interest to note that conditioning intensity (reduced‐intensity conditioning vs myeloablative *conditioning*) had no impact on outcomes of *TP53*‐mutant patients undergoing an allo‐SCT (Supporting Fig. 1).

In summary, this report provides the first comprehensive analysis of post‐transplant outcomes of patients with *TP53* mutant AML, because previous studies have mainly focused on the influence of *TP53* mutations, among other genetic factors, in patients with MDS.^8^, ^9^ These data suggest that patients with AML and *TP53* mutations without 17p– and/or CK should certainly remain transplant eligible. Even in the *TP53* mutant cohort with 17p– and/or CK (70% of patients in the *TP53* mutant cohort in this study), allo‐SCT still remains an important treatment strategy. These novel data are not only of immediate clinical relevance but also have importance for basic scientific studies focused on understanding the mechanism of *TP53* mutations.^35^

### FUNDING SUPPORT

This work was supported by research support and clinical trials funding from Cancer Research UK, Cure Leukemia, and Bloodwise.

### CONFLICT OF INTEREST DISCLOSURES

Gesine Bug reports a grant (paid to her institution) from Novartis; honoraria from Jazz Pharmaceuticals (paid to her institution), Celgene, and Gilead; support for attending meetings and/or travel from Jazz Pharmaceuticals, Gilead, and Neovii; and payment for participation on an advisory board from Novartis, Pfizer, Eurocept, Gilead, and Celgene. Charles Craddock reports grants from Celgene, Bloodwise, Cancer Research UK, and Cure Leukemia; and honoraria from Celgene, Daichi‐Sankyo, Novartis, and Pfizer. Justin Loke reports payment or honoraria from Pfizer, Janssen and Amgen; and travel support from Novartis and Daichi‐Sankyo. The other authors made no disclosures.

## AUTHOR CONTRIBUTIONS

**Justin Loke:** Study design, analysis, and writing. **Myriam Labopin:** Study design, analysis, and writing. **Charles Craddock:** Study design, analysis, and writing. **Arnon Nagler:** Study design, analysis, and writing. **Jordi Esteve:** Study design, analysis, and writing. **Mohamad Mohty:** Study design, analysis, and writing. **Jan J. Cornelissen:** Patient recruitment, study analysis, and writing. **Hélène Labussière‐Wallet:** Patient recruitment, study analysis, and writing. **Eva Maria Wagner‐Drouet:** Patient recruitment, study analysis, and writing. **Gwendolyn Van Gorkom:** Patient recruitment, study analysis, and writing. **Nicolaas P.M. Schaap:** Patient recruitment, study analysis, and writing. **Nicolaus M. Kröger:** Patient recruitment, study analysis, and writing. **Joan Hendrik Veelken:** Patient recruitment, study analysis, and writing. **Montserrat Rovira:** Patient recruitment, study analysis, and writing. **Anne Lise Menard:** Patient recruitment, study analysis, and writing. **Gesine Bug:** Patient recruitment, study analysis, and writing. **Ali Bazarbachi:** Patient recruitment, study analysis, and writing. **Sebastian Giebel:** Patient recruitment, study analysis, and writing. **Eolia Brissot:** Patient recruitment, study analysis, and writing.

## Supporting information


**Supporting information S1**Figure S1

**Supporting information S2**Table S1s

**Supporting information S3**Table S2
