# Supplementary material for: Additional cytogenetic features determine outcome in patients allografted for TP53 mutant acute myeloid leukemia
Source: Cancer. 2022 May 25;128(15):2922–31. doi: 10.1002/cncr.34268 (PMC9545190; doi:10.1002/cncr.34268)
Supplement: Supplementary file 1 — Supporting information S1Figure S1 [file CNCR-128-2922-s002.pptx]

## Slide 1
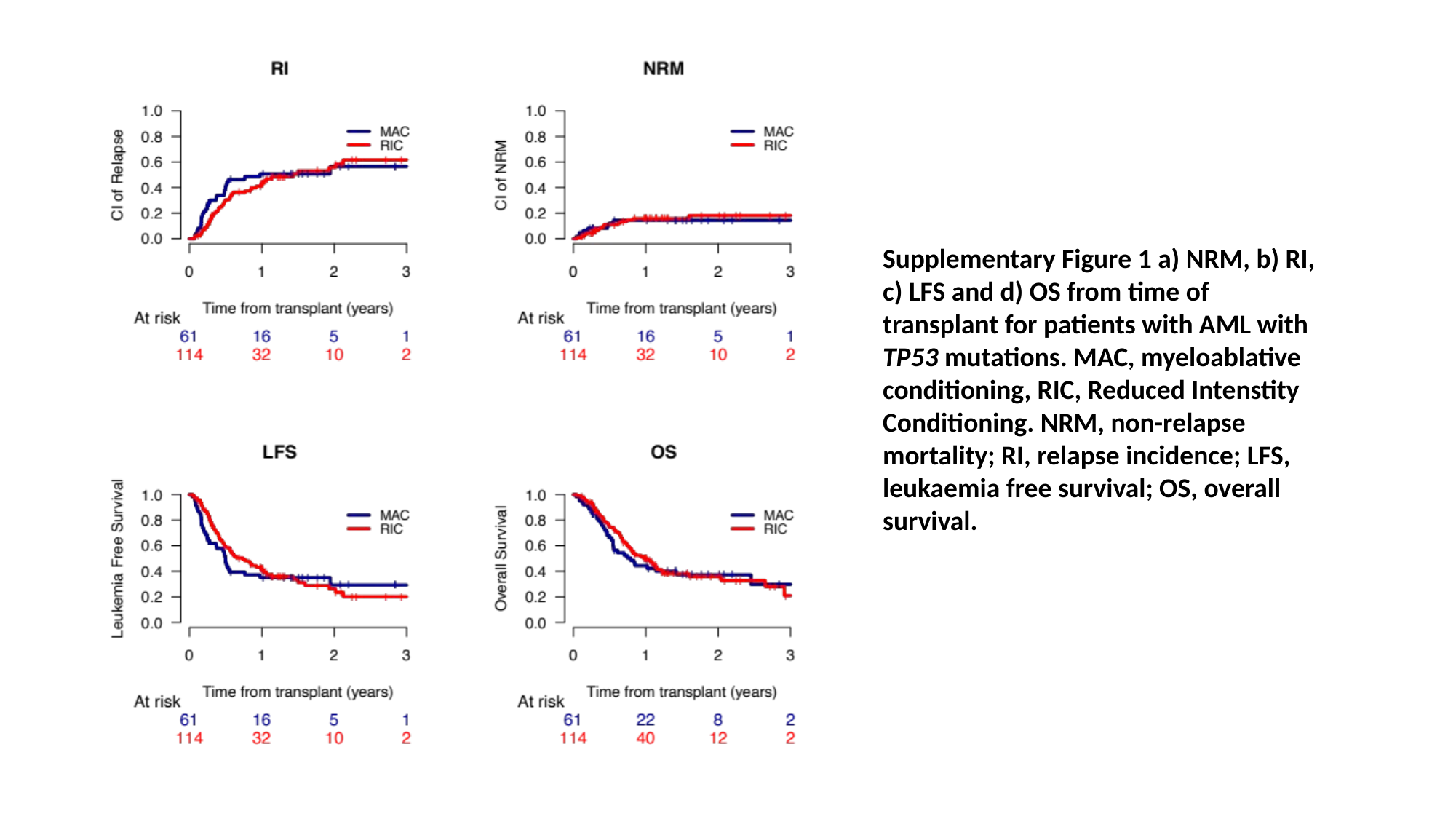

Supplementary Figure 1 a) NRM, b) RI, c) LFS and d) OS from time of transplant for patients with AML with TP53 mutations. MAC, myeloablative conditioning, RIC, Reduced Intenstity Conditioning. NRM, non-relapse mortality; RI, relapse incidence; LFS, leukaemia free survival; OS, overall survival.
