# Supplementary material for: Additional cytogenetic features determine outcome in patients allografted for TP53 mutant acute myeloid leukemia
Source: Cancer. 2022 May 25;128(15):2922–31. doi: 10.1002/cncr.34268 (PMC9545190; doi:10.1002/cncr.34268)
Supplement: Supplementary file 3 — Supporting information S3Table S2 [file CNCR-128-2922-s003.docx]

**Supplementary table 2 Univariate analysis of transplant outcomes at 2 years.** Estimates [95% CI], Non-relapse mortality (NRM), Leukaemia free survival (LFS), Overall Survival (OS) (next page).

|  |  | **Relapse** | **NRM** | **LFS** | **OS** | **Acute GVHD II-IV** | **ext. chronic GVHD** |
| --- | --- | --- | --- | --- | --- | --- | --- |
| *TP53* mutation | Absent | 25.2%[21.2-29.3] | 17%[13.8-20.5] | 57.8%[53.1-62.3] | 64%[59.1-68.4] | 29.7%[26-33.5] | 18.3%[14.7-22.2] |
|  | Present | 55%[45.2-63.8] | 17.7%[11.7-24.6] | 27.3%[19-36.3] | 35.1%[26.7-43.7] | 32.1%[25.3-39.2] | 15.6%[9.8-22.7] |
|  | P value | 0.001 | 0.96 | 0.001 | 0.001 | 0.53 | 0.41 |
| Complex Karyotype | Absent | 24.5%[20.5-28.7] | 17.3%[14-20.8] | 58.2%[53.4-62.7] | 64.9%[60-69.3] | 29.6%[25.9-33.4] | 17.9%[14.3-21.9] |
|  | Present | 53.6%[45-61.5] | 16.5%[11.1-22.9] | 29.9%[22.4-37.8] | 35.5%[27.6-43.6] | 32.4%[25.7-39.3] | 17.2%[11.4-24.1] |
|  | P value | 0.001 | 0.78 | 0.001 | 0.001 | 0.44 | 0.82 |
| Monosomal Karyotype | Absent | 26.3%[22.4-30.3] | 17.8%[14.7-21.2] | 55.9%[51.3-60.2] | 60.9%[56.3-65.2] | 30.6%[27-34.2] | 19.3%[15.8-23.1] |
|  | Present | 58.8%[47.8-68.2] | 13.2%[7.4-20.7] | 28%[19-37.8] | 40.6%[30.2-50.8] | 28.9%[20.9-37.2] | 9.5%[4.6-16.5] |
|  | P value | 0.001 | 0.16 | 0.001 | 0.001 | 0.67 | 0.06 |
| Abnormal 17p | Absent | 28.5%[24.6-32.4] | 16.8%[13.9-20] | 54.7%[50.3-58.9] | 60.8%[56.3-65] | 30%[26.6-33.4] | 18.5%[15.2-22.1] |
|  | Present | 62.1%[47.3-73.8] | 19.2%[9.9-30.7] | 18.8%[9.3-30.8] | 25.1%[14.3-37.5] | 33.5%[22.8-44.5] | 10.4%[3.7-21.1] |
|  | P value | 0.001 | 0.8 | 0.001 | 0.001 | 0.56 | 0.11 |
| Del(7q)/-7 | Absent | 28.1%[24.2-32.1] | 16.6%[13.6-19.9] | 55.3%[50.8-59.6] | 61.2%[56.6-65.4] | 29.9%[26.4-33.4] | 17.6%[14.2-21.3] |
|  | Present | 51.7%[40.1-62.2] | 20.1%[12.2-29.3] | 28.2%[18.4-38.8] | 37.4%[26.6-48.2] | 32.9%[24-42] | 18.2%[10.9-27.1] |
|  | P value | 0.001 | 0.59 | 0.001 | 0.001 | 0.57 | 0.74 |
| Del(5q)/-5 | Absent | 26%[22.2-30] | 16.8%[13.8-20.1] | 57.2%[52.7-61.5] | 63.1%[58.5-67.4] | 29.7%[26.2-33.2] | 19.1%[15.6-22.9] |
|  | Present | 64.5%[52.4-74.3] | 19.2%[11.7-28.2] | 16.2%[8.5-26.2] | 25.7%[16.7-35.7] | 34%[25.2-43] | 9.3%[4.3-16.8] |
|  | P value | 0.001 | 0.9 | 0.001 | 0.001 | 0.39 | 0.023 |
| AML type | Primary | 30.6%[26.5-34.8] | 16.3%[13.3-19.6] | 53.1%[48.5-57.5] | 58.5%[53.8-62.9] | 30.6%[27.1-34.2] | 18.1%[14.6-21.8] |
|  | Secondary AML | 36.3%[26.6-46.1] | 21.7%[14.3-30] | 42%[31.9-51.7] | 51.9%[40.8-62] | 28.5%[20.3-37.2] | 16.3%[9.2-25.1] |
|  | P value | 0.18 | 0.1 | 0.009 | 0.1 | 0.73 | 0.61 |
| Type of donor | Matched sibling | 35.6%[28-43.3] | 9.9%[5.6-15.8] | 54.5%[45.9-62.3] | 58.8%[49.8-66.8] | 29.3%[23-35.9] | 20.8%[14.4-28.1] |
|  | Unrelated | 30.9%[25.9-35.9] | 18.8%[15-22.9] | 50.3%[44.8-55.6] | 56.7%[51.1-61.9] | 31.2%[27-35.5] | 18.3%[14.3-22.8] |
|  | Other | 28%[19-37.6] | 21.6%[14.4-29.8] | 50.4%[39.8-60.1] | 59.7%[49.2-68.6] | 28.4%[20.5-36.9] | 10.4%[4.9-18.3] |
|  | P value | 0.34 | 0.003 | 0.34 | 0.19 | 0.81 | 0.052 |
| Conditioning intensity | MAC | 31.8%[25.8-37.9] | 13.4%[9.7-17.7] | 54.8%[48.1-61] | 60.4%[53.5-66.6] | 32.1%[27-37.4] | 15.1%[10.7-20.2] |
|  | RIC | 31.3%[26.4-36.3] | 19.4%[15.4-23.6] | 49.3%[43.8-54.6] | 55.8%[50.1-61] | 28.8%[24.7-33.1] | 19.6%[15.4-24.2] |
|  | P value | 0.75 | 0.056 | 0.3 | 0.15 | 0.27 | 0.28 |
| Karnofsky score | <90 | 31.2%[24-38.6] | 20.3%[14.5-26.8] | 48.5%[40.4-56.2] | 55.1%[46.5-62.8] | 34.5%[27.6-41.5] | 13.8%[8.9-19.9] |
|  | >=90 | 31.1%[26.5-35.7] | 15.3%[12.1-18.8] | 53.7%[48.6-58.5] | 59.6%[54.5-64.4] | 29.9%[26.1-33.7] | 19.5%[15.6-23.8] |
|  | P value | 1 | 0.17 | 0.25 | 0.56 | 0.26 | 0.25 |
| In vivo T-cell depletion | Absent | 29.7%[24.5-35.2] | 17.5%[13.4-22] | 52.8%[46.7-58.5] | 58%[51.9-63.7] | 31.8%[27.1-36.7] | 22.7%[17.7-28] |
|  | Present | 33.8%[28.3-39.3] | 17.1%[13.1-21.5] | 49.1%[43.1-54.9] | 56.5%[50.3-62.2] | 28.7%[24.3-33.3] | 13.3%[9.7-17.6] |
|  | P value | 0.27 | 0.66 | 0.56 | 0.95 | 0.34 | 0.003 |

**Supplementary table 3: Univariate analysis of factors that influence outcomes of patients with TP53 mutated AML**

|  |  | **Outcomes at 18 months** | | | | **180 days** | **18 months** |
| --- | --- | --- | --- | --- | --- | --- | --- |
|  |  | **Relapse** | **NRM** | **LFS** | **OS** | **Acute GVHD II-IV** | **chronic GVHD** |
| Patient age | age<=60y (median) | 46.2%[34.2-57.2] | 10.7%[4.9-19] | 43.2%[31.5-54.3] | 46.6%[34.3-58] | 25.1%[16.5-34.6] | 31.5%[21.1-42.4] |
|  | age>60y | 58.5%[43.8-70.6] | 21.8%[13.2-31.8] | 19.7%[9.9-32] | 28.8%[18.3-40.3] | 39.3%[28.9-49.5] | 20.6%[11.5-31.6] |
|  | P value | 0.55 | 0.024 | 0.011 | 0.003 | 0.06 | 0.09 |
|  |  |  |  |  |  |  |  |
| Type of AML | de novo | 48.8%[38.8-58] | 15.6%[9.7-22.7] | 35.7%[26.5-44.9] | 37.3%[28-46.6] | 34.6%[26.8-42.5] | 28.8%[20.5-37.6] |
|  | Secondary AML | 61%[37.8-77.8] | 19%[7.4-34.5] | 20.1%[6.9-38] | 40.1%[22.6-57] | 21.4%[9.2-36.8] | 17.2%[6-33.3] |
|  | P value | 0.2 | 0.68 | 0.07 | 0.49 | 0.14 | 0.25 |
|  |  |  |  |  |  |  |  |
| Del(5q)/-5 | Absent | 39.4%[27.8-50.8] | 15.9%[8.9-24.8] | 44.7%[32.7-56] | 51.8%[39.6-62.7] | 31.9%[22.5-41.6] | 36.2%[25.2-47.2] |
|  | Present | 65.1%[50.5-76.4] | 16.6%[8.9-26.3] | 18.3%[9-30.2] | 22.5%[12.7-33.9] | 32.5%[22.7-42.7] | 15.8%[7.9-26.2] |
|  | P value | 0.001 | 0.86 | 0.001 | 0.001 | 0.87 | 0.006 |
|  |  |  |  |  |  |  |  |
| Abnormal 17p | Absent | 44.6%[33.8-54.8] | 16%[9.7-23.8] | 39.4%[29-49.6] | 47.1%[36.6-56.8] | 31.8%[23.7-40.3] | 29.4%[20.5-38.8] |
|  | Present | 66.9%[50.1-79.2] | 16.2%[7.5-28] | 16.8%[7.2-29.9] | 19%[8.8-32.2] | 32.7%[20.7-45.3] | 19.4%[8.6-33.4] |
|  | P value | 0.007 | 0.65 | 0.001 | 0.001 | 0.88 | 0.21 |
|  |  |  |  |  |  |  |  |
| Del(7q)/-7 | Absent | 44.5%[33.9-54.6] | 16.4%[9.9-24.3] | 39%[28.8-49.1] | 44.3%[34-54.2] | 32.8%[24.4-41.4] | 25.9%[17.4-35.2] |
|  | Present | 64.4%[47.3-77.2] | 15.7%[7.2-27.3] | 19.9%[9.1-33.5] | 27.2%[15-40.9] | 31%[19.6-43.2] | NA |
|  | P value | 0.058 | 0.94 | 0.06 | 0.12 | 0.83 | 0.91 |
|  |  |  |  |  |  |  |  |
| Complex Karyotype | Absent | 30.8%[17.2-45.5] | 14.1%[6.5-24.7] | 55%[38.8-68.6] | 61.9%[46.8-73.8] | 31.1%[19.9-42.9] | 29.1%[17-42.4] |
|  | Present | 66.4%[54.6-75.8] | 19%[11.6-27.8] | 14.5%[7.4-23.9] | 23.1%[14.7-32.8] | 32.8%[24.3-41.5] | 24.7%[16.2-34.2] |
|  | P value | 0.001 | 0.69 | 0.001 | 0.001 | 0.86 | 0.55 |
|  |  |  |  |  |  |  |  |
| Monosomal Karyotype | Absent | 43.4%[31.6-54.7] | 17.8%[10.6-26.5] | 38.8%[27.4-50] | 46.1%[34.9-56.5] | 33%[24.1-42.2] | 29.6%[20-39.9] |
|  | Present | 69.8%[53.9-81.1] | 16.6%[8.1-27.8] | 13.6%[5.2-26] | 22.5%[12.1-34.9] | 31%[20.6-41.9] | 21.4%[11.7-33.2] |
|  | P value | 0.003 | 0.76 | 0.005 | 0.016 | 0.76 | 0.26 |
|  |  |  |  |  |  |  |  |
| Patient sex | Male | 50.9%[38.7-61.8] | 19.9%[11.9-29.4] | 29.3%[19.2-40.1] | 36.7%[25.6-47.8] | 28.1%[19.1-37.7] | 16.1%[8.7-25.4] |
|  | Female | 51%[37.5-62.9] | 12.2%[5.9-21] | 36.8%[24.7-49] | 39.7%[27.4-51.6] | 36.5%[26.4-46.7] | 38.4%[26.4-50.2] |
|  | P value | 0.82 | 0.27 | 0.45 | 0.5 | 0.15 | 0.01 |
|  |  |  |  |  |  |  |  |
| Type of donor | Matched Sibling | 50.2%[30.9-66.8] | 9.9%[2.4-24.1] | 39.8%[22.5-56.7] | 37.7%[18.3-57.1] | 24.3%[11.9-39.1] | 36.2%[19.2-53.4] |
|  | Unrelated donor | 53.6%[41.8-63.9] | 15.6%[9.1-23.8] | 30.8%[20.8-41.3] | 35.7%[25.4-46.1] | 32.9%[24.2-41.8] | 24.2%[15.4-34] |
|  | Other | 42.3%[21.4-61.9] | 27.3%[11.6-45.8] | 30.4%[13.3-49.5] | 42.9%[22.9-61.5] | 39.3%[21.2-56.9] | 21.9%[7.4-41.2] |
|  | P value | 0.66 | 0.16 | 0.68 | 0.53 | 0.44 | 0.22 |
|  |  |  |  |  |  |  |  |
| Conditioning | MAC | 50.7%[36.3-63.4] | 14.3%[6.6-25] | 35%[22.3-47.9] | 37.2%[23.9-50.5] | 25.5%[15.1-37.2] | 22.4%[11.9-35.1] |
|  | RIC | 53.1%[40.9-64] | 15.7%[9.2-23.9] | 31.2%[20.7-42.1] | 38.1%[27.4-48.7] | 35%[26.3-43.9] | 28.8%[19.3-39] |
|  | P value | 0.41 | 0.86 | 0.38 | 0.64 | 0.25 | 0.49 |
|  |  |  |  |  |  |  |  |
| Karnofsky score | <90 | 54.6%[35.4-70.3] | 13.4%[5.3-25.3] | 32%[16.7-48.3] | 37.3%[21.7-52.9] | 31.9%[19.1-45.5] | 24.8%[12.1-39.8] |
|  | >=90 | 49.1%[38.2-59.1] | 16.5%[10-24.4] | 34.4%[24.6-44.5] | 39.6%[29.4-49.6] | 34.7%[26.2-43.3] | 28.9%[20-38.4] |
|  | P value | 0.91 | 0.94 | 0.86 | 0.92 | 0.7 | 0.41 |
|  |  |  |  |  |  |  |  |
| Patient CMV | CMV neg. | 51.8%[38.2-63.7] | 12.5%[5.7-22] | 35.7%[23.7-47.9] | 38.6%[25.1-51.9] | 31.1%[20.7-42.1] | 19%[10-30.2] |
|  | CMV pos | 50.2%[38.4-61] | 18.8%[11.4-27.6] | 31%[20.8-41.8] | 37.4%[27-47.9] | 32.8%[23.9-42] | 31.4%[21.5-41.8] |
|  | P value | 0.34 | 0.28 | 0.87 | 0.75 | 0.84 | 0.12 |
|  |  |  |  |  |  |  |  |
| Donor CMV | CMV neg. | 57.5%[44.2-68.7] | 19.3%[11.3-28.9] | 23.3%[13.6-34.5] | 28.5%[18.2-39.8] | 33.1%[23.4-43] | 22.1%[13.3-32.3] |
|  | CMV pos | 44.5%[31.9-56.3] | 13.2%[6.6-22.1] | 42.3%[30-54.1] | 48.5%[35.8-60] | 31.6%[22-41.6] | 31.2%[20.2-42.8] |
|  | P value | 0.16 | 0.4 | 0.028 | 0.06 | 0.71 | 0.45 |
|  |  |  |  |  |  |  |  |
| In vivo T-cell depletion (TCD) | no in vivo TCD | 51.7%[38.4-63.5] | 17.9%[10-27.6] | 30.4%[19.3-42.3] | 35.3%[23.3-47.4] | 37.5%[26.9-48] | 27.8%[17.4-39.2] |
|  | in vivo TCD | 51.3%[38.7-62.6] | 14.1%[7.4-23] | 34.6%[23.4-46] | 40.4%[29-51.5] | 28%[19.2-37.4] | 25.3%[15.8-35.8] |
|  | P value | 0.72 | 0.32 | 0.67 | 0.86 | 0.22 | 0.73 |
|  |  |  |  |  |  |  |  |
| Post-transplant cyclophosphamide (PTCy) | no PTCy | 54.8%[44.2-64.2] | 15.5%[9.4-23.1] | 29.7%[20.7-39.2] | 34.2%[24.9-43.8] | 30.5%[22.8-38.6] | 25.7%[17.6-34.5] |
|  | PTCy | 45%[26.3-62.1] | 18.2%[7.9-32] | 36.7%[20-53.5] | 44.4%[26.6-60.9] | 40%[24.8-54.8] | 28.1%[13.5-44.6] |
|  | P value | 0.041 | 0.39 | 0.14 | 0.19 | 0.32 | 0.96 |
|  |  |  |  |  |  |  |  |
| MRD before HSCT | MRD neg | 40.4%[20-60] | 3.9%[0.3-17.2] | 55.7%[33.3-73.2] | 64.5%[41.5-80.4] | 20.1%[8.7-34.9] | 34%[16.3-52.7] |
|  | MRD pos | 59%[36.8-75.6] | 14.7%[5.2-28.8] | 26.4%[11.3-44.2] | 37.3%[20.3-54.4] | 41.7%[25.3-57.2] | 23%[9.7-39.5] |
|  | P value | 0.11 | 0.13 | 0.011 | 0.008 | 0.05 | 0.3 |

**Supplementary table 4: Cox multivariate analysis of variables which affect outcomes of patients with *TP53* mutated AML**

|  | **Relapse** | | **NRM** | | **LFS** | | **OS** | | **Acute GVHD II-IV** | | **Chronic GVHD** | |
| --- | --- | --- | --- | --- | --- | --- | --- | --- | --- | --- | --- | --- |
|  | HR (95% CI) | p value | HR (95% CI) | p value | HR (95% CI) | p value | HR (95% CI) | p value | HR (95% CI) | p value | HR (95% CI) | p value |
| Abnormal 17p | 1.62 (0.98-2.69) | 0.06 | 1.46 (0.59-3.6) | 0.42 | 1.59 (1.02-2.48) | 0.039 | 1.71 (1.08-2.71) | 0.023 | 0.99 (0.53-1.84) | 0.97 | 0.87 (0.37-2.09) | 0.76 |
| Del(5q)/-5 | 1.45 (0.84-2.51) | 0.18 | 1.44 (0.52-3.96) | 0.49 | 1.42 (0.88-2.28) | 0.15 | 1.39 (0.83-2.34) | 0.21 | 0.99 (0.49-1.99) | 0.98 | 0.48 (0.2-1.15) | 0.099 |
| Complex Karyotype | 2.55 (1.25-5.18) | 0.01 | 1.33 (0.44-4.07) | 0.62 | 2.11 (1.17-3.82) | 0.013 | 1.99 (1.06-3.75) | 0.033 | 1.14 (0.56-2.34) | 0.72 | 1.54 (0.64-3.74) | 0.34 |
| Monosomal Karyotype | 0.88 (0.5-1.55) | 0.66 | 0.61 (0.21-1.77) | 0.36 | 0.8 (0.49-1.32) | 0.39 | 0.71 (0.41-1.23) | 0.22 | 0.78 (0.38-1.59) | 0.5 | 1.21 (0.47-3.07) | 0.69 |
| Age (per 10 y) | 0.93 (0.75-1.15) | 0.5 | 1.4 (0.89-2.18) | 0.14 | 1.02 (0.84-1.23) | 0.83 | 1.13 (0.92-1.38) | 0.25 | 1.36 (1.03-1.8) | 0.032 | 0.77 (0.57-1.05) | 0.1 |
| secondary AML vs de novo AML | 1.7 (1-2.9) | 0.051 | 1.36 (0.52-3.52) | 0.53 | 1.63 (1.03-2.6) | 0.039 | 1.17 (0.7-1.95) | 0.55 | 0.54 (0.24-1.22) | 0.14 | 0.72 (0.25-2.08) | 0.54 |
| Patient CMV pos | 1.29 (0.75-2.21) | 0.35 | 1.87 (0.73-4.81) | 0.19 | 1.39 (0.88-2.22) | 0.16 | 1.47 (0.91-2.39) | 0.12 | 1.09 (0.59-2.03) | 0.78 | 2.11 (0.9-4.91) | 0.084 |
| Donor CMV pos | 0.64 (0.38-1.06) | 0.084 | 0.51 (0.21-1.24) | 0.14 | 0.6 (0.39-0.93) | 0.024 | 0.63 (0.39-1) | 0.05 | 0.73 (0.4-1.34) | 0.3 | 0.65 (0.3-1.41) | 0.27 |
| Post-transplant cyclophosphamide | 0.63 (0.35-1.16) | 0.14 | 1.07 (0.44-2.61) | 0.89 | 0.74 (0.45-1.21) | 0.23 | 0.72 (0.43-1.21) | 0.22 | 1.19 (0.65-2.17) | 0.56 | 0.78 (0.35-1.73) | 0.54 |
